# Sensory and Emotional Perception of Wooden Surfaces through Fingertip Touch

**DOI:** 10.3389/fpsyg.2017.00367

**Published:** 2017-03-13

**Authors:** Shiv R. Bhatta, Kaisa Tiippana, Katja Vahtikari, Mark Hughes, Marketta Kyttä

**Affiliations:** ^1^Department of Built Environment, School of Engineering, Aalto UniversityEspoo, Finland; ^2^Department of Psychology and Logopedics, Faculty of Medicine, University of HelsinkiHelsinki, Finland; ^3^Department of Bioproducts and Biosystems, Aalto UniversityEspoo, Finland

**Keywords:** emotional touch, tactile perception, fingertip touch, texture perception, wood, surface treatments

## Abstract

Previous studies on tactile experiences have investigated a wide range of material surfaces across various skin sites of the human body in self-touch or other touch modes. Here, we investigate whether the sensory and emotional aspects of touch are related when evaluating wooden surfaces using fingertips in the absence of other sensory modalities. Twenty participants evaluated eight different pine and oak wood surfaces, using sensory and emotional touch descriptors, through the lateral motion of active fingertip exploration. The data showed that natural and smooth wood surfaces were perceived more positively in emotional touch than coated surfaces. We highlight the importance of preserving the naturalness of the surface texture in the process of wood-surface treatment so as to improve positive touch experiences, as well as avoid negative ones. We argue that the results may offer possibilities in the design of wood-based interior products with a view to improving consumer touch experiences.

## Introduction

The tactile experiences of material surfaces originate in parts of the sensory and emotional touch sensations felt in our skin (McGlone et al., 2007), where the former relates to the discriminative functions of touch associated with the perceptions of pressure, vibration, slip, and texture, and the latter to the positive and negative affective aspects of the cutaneous sensation (Vallbo et al., 1993, 1999; Mountcastle, 2005; McGlone et al., 2012, 2014). Typically, the conscious touch of physical surface properties and subjective feelings emerge hand-in-hand during the evaluation of an object through the sense of touch. The importance of the emotional aspect of touch in consumer behavior covers a range of matters like preferences, well-being, and satisfaction in product purchase (Berger et al., 2006; Khalid and Helander, 2006). There have been numerous attempts made to understand the influence of the sensory properties of material surfaces on emotional touch evaluation (McGlone et al., 2007, 2012; Guest et al., 2011; Ackerley et al., 2014a; Fujisaki et al., 2015). To date however, there have been few studies of the material-specific and fully tactile experiences of surfaces that have the potential to guide the design aspect of consumer products with a view to improving consumer touch experiences. Wood, being perceived as natural and eco-friendly, is currently attracting much interest as a material for living spaces, and thus a study on the tactile experience of wood surfaces is timely. In the study reported here, we investigated whether the sensory and emotional perception of wooden surfaces are related and, if so, how this might offer possibilities to improve the positive touch experiences of wood products.

Efforts have been made to discriminate between the perception of the properties of material surfaces by covering a breadth of physical parameters including roughness, hardness (Lederman and Klatzky, 1987, 2009; LaMotte, 2000; Guest and Spence, 2003), slipperiness (Hollins et al., 2000), as well as firmness, and pile (Guest et al., 2011). Similarly, a wide range of adjectives, such as prettiness, expensiveness, naturalness, likableness, pleasantness, and relaxed as well as interesting, have been used to assess emotional touch aspects (Overvliet and Soto-Faraco, 2011; Fleming et al., 2013; Fujisaki et al., 2015). Guest et al. (2011) argue that little is known about the importance of these adjectives when representing emotional touch, except for pleasantness and associated perceptions such as comfort (Essick et al., 2010), and suggested a Touch Perception Task (TPT) having separate lists for sensory and emotional touch descriptors. Using Guest et al.'s TPT descriptors, McGlone et al. (2012) and Ackerley et al. (2014a) reported similar results in psychophysical observations. Additionally, a number of neurobehavioral studies on tactile perception have suggested that there is some association between the discriminative and emotional aspects of touch, depending on the touch mode (active or passive touch), skin sites (e.g., forearm, palm, face), skin condition (hairy or non-hairy skin) and the types of material surfaces touched (McGlone et al., 2007, 2012; Ackerley et al., 2014a). A recent study in which 22 varieties of genuine and fake wood surfaces were assessed with various combinations of touch, vision, and hearing also suggested an association between sensory and affective aspects (Fujisaki et al., 2015). Most affective adjectives used by Fujisaki et al. (2015) referred to preferential and evaluative characteristics, such as cheap–expensive, boring–interesting, dislike–like, these representing the overall significance and importance of touch experiences. In the present study, we attempt to be more specific in defining the emotional touch descriptors that refer to “the feelings that only occurred when touching or being touched” (Guest et al., 2011).

Another aspect of the tactile experience is the existence of separate neural mechanisms underlying sensory and emotional touch in the human body (Olausson et al., 2002; McGlone et al., 2007, 2014; Löken et al., 2011; Gordon et al., 2013). The A-beta afferent fibers are dedicated to conveying the discriminative aspect of touch (McGlone and Reilly, 2010; Kandel et al., 2013), whereas C-tactile afferents are thought to be responsible for emotional touch but are absent from glabrous skin (Olausson et al., 2010). Thus any aspect of pleasantness during touch with the hands must be signaled by A-beta afferents (Klöcker et al., 2012; McGlone et al., 2012). Research suggests that the nature and intensity of the affective touch perception originating in glabrous skin is different to that of hairy skin. For example, soft brush strokes are perceived to be less pleasant on the palm than on a hairy arm (Löken et al., 2011; Ackerley et al., 2014b). However, where there has been prior stimulation of the arm, or where the stimulation is ongoing, a greater degree of pleasantness is perceived on the palm, though not vice versa (Löken et al., 2011). On the other hand, the glabrous skin of the hand provides opportunities for active exploration of the environment and serves as the main source of tactile input to the brain for evaluative touch. The higher level of cognitive engagement in affective perception induced by A-beta stimulation, in which previous affect inputs (originating from either glabrous or from hairy skin) serve as unconscious affective cues, should support product touch evaluation (Löken et al., 2011). This kind of “priming” effect has already been reported with visual perception, where affect-embedded visual information has been shown to influence the affective reaction to food (Berridge, 2003; Gibbons, 2009). It seems that either the sensory or the affective nature of previous tactile inputs in both types of skin should enhance the evaluation of products by touch using hands.

It is important to be aware of the extent to which glabrous skin is limited by its ability to perceive the emotional aspects of touch despite its supremacy in discriminative sensation (Craig and Lyle, 2001; Lederman and Klatzky, 2009). There are a number of contextual factors affecting the intensity of the evoked sensation such as skin sites, the speed of stroking, and self-touch vs. other touch modes (Guest et al., 2011; Ackerley et al., 2014a). A soft gentle stroking of the skin at a speed of 1–10 cm/s, when touched by another person, is perceived to be the most pleasant touch type on both hairy skin, for example on the arm, and on glabrous skin, for example on the palm (Löken et al., 2009; Ackerley et al., 2014b). Furthermore, Perini et al. (2015) found that the preferred speeds of stroking on the arm are 1, 3, and 10 cm/s but on the palm it is 3 cm/s, suggesting a narrower range of stroking speed for the pleasantness perception through glabrous skin. In addition, a lateral motion of the hand over a surface is essential for optimal performance in surface-texture perception in the haptic mode (Lederman and Klatzky, 2009), meaning that haptic factors other than texture (such as shape, weight, compliance, and temperature) are less important when hand movement is applied in this kind of surface exploration.

There has been much effort directed at trying to identify the breadth of the discriminative properties in material surface perception. Amongst these, rough-smooth (Hollins et al., 1993, 2000) and soft-hard (LaMotte, 2000) perceptions have been identified as primary dimensions, independently available to the touch sense (for more details of tactile dimensions, see Okamoto et al., 2013). Additionally, sticky-slippery (Hollins et al., 2000), hot-cold and dry-wet (Guest et al., 2011) perceptions have been reported to be secondary dimensions in the discriminative perception of texture. However, the soft-hard, and hot-cold dimensions are not important in the lateral finger movement method of texture exploration over plain surfaces (Berger and Salvenmoser, 2006). Whether the slippery-sticky and moist-dry dimensions come from a single dimension, termed the frictional dimension (Okamoto et al., 2013), or emerge independently (Shirado and Maeno, 2005; Yoshioka et al., 2007) is controversial.

The aim of the present study is to investigate the sensory and emotional perception of touch across a variety of wooden surfaces through active fingertip exploration in the absence of vision. Since the underlying relationship between the tactile experience and the texture of the wood surface is crucial in the design of wooden products (such as furniture or interior spaces), our aim is also to explore how the sensory and emotional aspects of touch are related. To investigate this empirically, we created eight types of surfaces from Scots pine and oak wood boards by applying four types of treatments to each species. A touch questionnaire in the Finnish language was used to assess eight emotional and three sensory characteristics of tactile experiences. In addition, sandpaper, and silk cloth were included as reference stimuli to examine whether they were perceived in a similar way to previous studies that included these media (sandpaper was used by Ackerley et al., 2014a and silk cloth by Guest et al., 2011). The findings are further discussed in relation to possible applications in the design of wooden products to maximize the positive aspects of product-touch experiences.

## Materials and methods

### Participants

Twenty healthy participants aged 19–26 years (mean age = 21.8 years, *SD* = 1.98; 8 male and 12 female), amongst which two male and two female were left-handed, took part in the experiment. All participants were native Finnish-speaking students and were novices with regard to this experiment. Each participant took <90 min to complete the experiment and was reimbursed with two movie tickets. Written information was provided prior to the experiment and all the participants gave informed consent. The study was approved by the University Ethics Committee of Aalto University, Finland and was conducted in accordance with the Declaration of Helsinki.

### Touch stimuli

**Figure 1:**
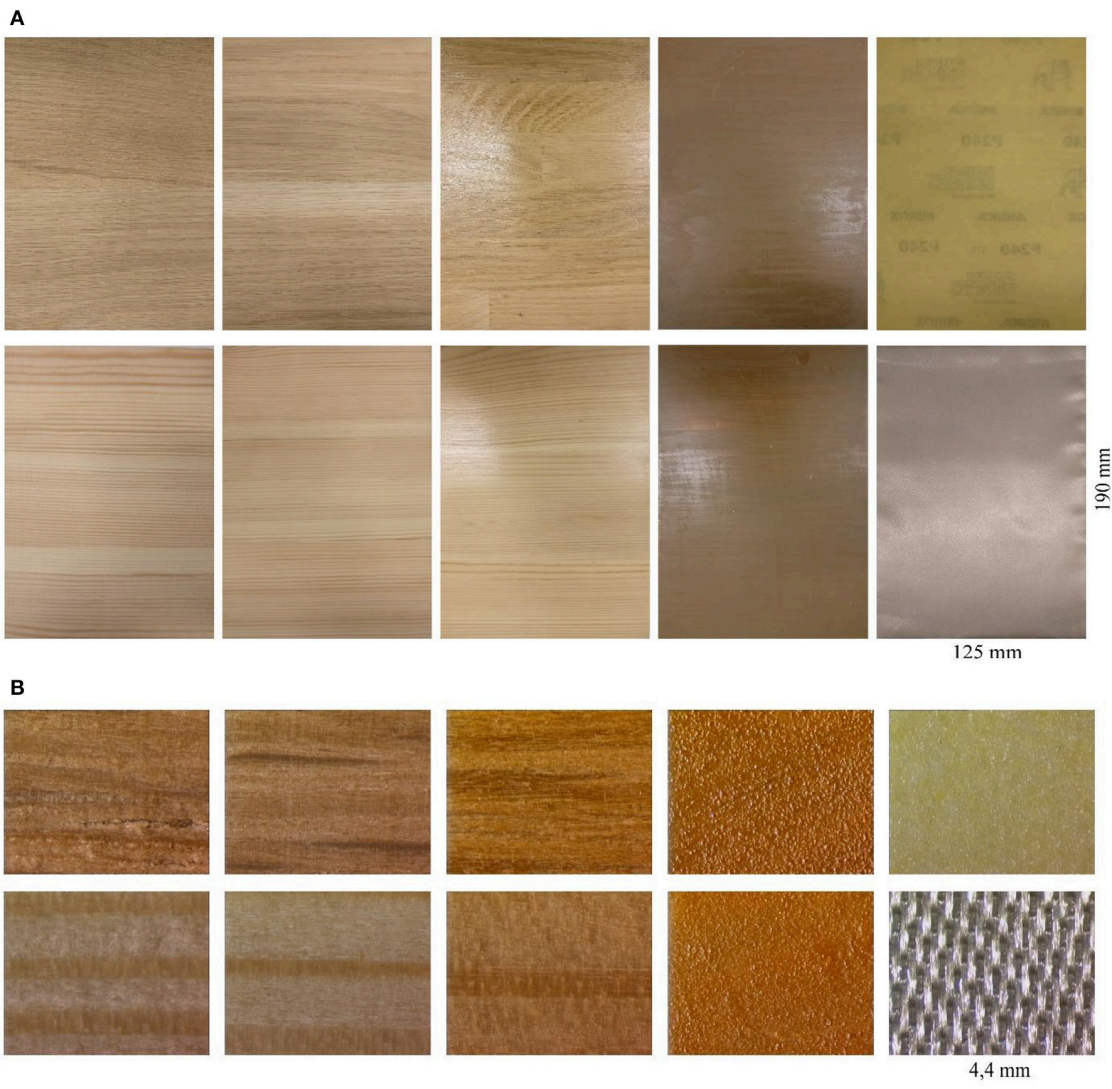
**Test stimuli: (A)**. Images under normal camera **(B)**. Microscopic images shown under magnifying area of a 4 mm surface length. Order of the images in figures **(A,B)** from left to right: First row includes brushed oak, sanded oak, varnished oak, waxed oak, and sandpaper-240. Second row includes brushed pine, sanded pine, varnished pine, waxed pine, and silk cloth.

The wood species used in the study were Scots pine (*Pinus sylvestris* L.) and oak (*Quercus robur* L.), representing softwoods and hardwoods, respectively. The stimuli were prepared by gluing together three small pieces of wood, each with a similar surface appearance, and then machine sanding the assembly to create a uniform surface. The sizes of the stimuli were identical: 20 cm long, 20 cm wide and 1.7 cm thick. Four stimuli types were prepared from each species. The surfaces were first sanded with 60-grit sandpaper after which the four surface treatments were created as follows: (1) by sanding with 240-grit sandpaper; (2) by brushing with a metal-brush; (3) by applying two coats of varnish; and (4) by the application of two layers of wax (Table 1, Figure 1). The varnish was applied by brush and the wax with a piece of cotton cloth. In addition, two further stimuli were prepared as references by attaching 240-grit sandpaper and silk cloth to wood boards having the same dimensions as those of the wood stimuli.

**Table 1:** **Stimuli descriptions and assigned ID used in the experiment**.

**Sample No**	**Sample ID**	**Descriptions**
1	PR1	Pine surface sanded with sandpaper-240
2	PR2	Pine surface sanded with metal brush
3	PV	Pine surface coated with double layer varnish
4	PW	Pine surface coated with double layer wax
5	OR1	Oak surface sanded with sandpaper-240
6	OR2	Oak surface sanded with metal brush
7	OV	Oak surface coated with double layer varnish
8	OW	Oak surface coated with double layer wax
9	SLK	Silk cloth^*^
10	SP240	Sandpaper-240^*^
11	A4 Paper	For practicing hand movement^**^

* *SLK and SP240 were prepared by wrapping the materials on wood boards of similar dimensions with other wood stimuli*.

** *A4 paper was used only for practicing the hand movement*.

### Experimental setup

**Figure 2:**
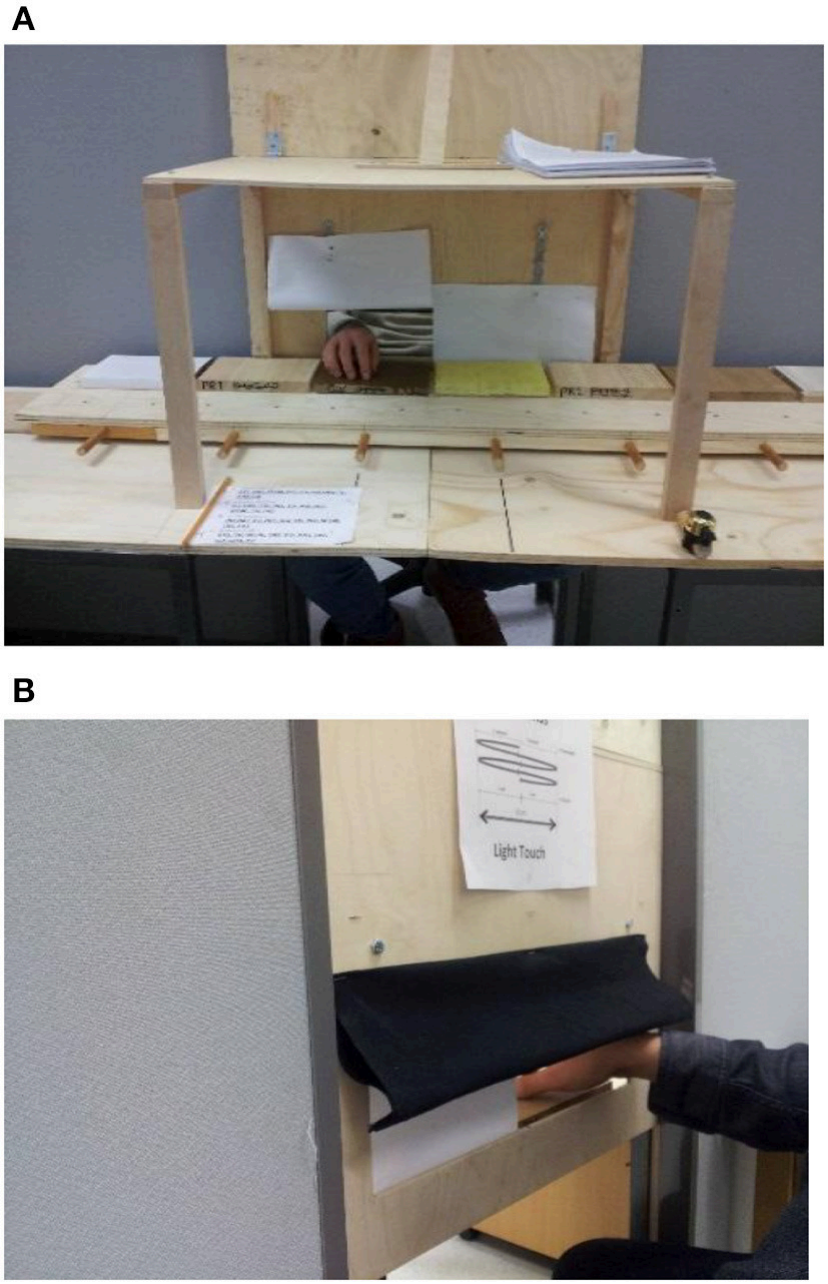
**Experimental setup: (A)**. Experimenter's side view **(B)**. Participant's side view.

A custom-designed setup (Figure 2) was used for the experiment. The apparatus consisted of a long table with a movable surface on the top. The touch-stimuli were located on the movable surface. The front view from the participant's side was completely blocked off, and a 50 × 10 cm rectangular opening was made for the hand to be inserted to touch the stimulus surface. The opening was equally accessible to both hands. Paper curtains were fixed on the inner side of the opening and a movable cloth on the outer side, which was adjusted according to the height of the participant to prevent an accidental sight of the stimulus surface. In addition, the experimenter was able to see the participant's hand and was therefore able to correct any error during the trial by asking for repetition, if needed. The participants sat on a height-adjustable chair during the trials, and a table for writing the responses was located either on the left or the right side of the participant according to their dominant hand (the dominant hand was used for touch purposes). Bells were provided for both the participant and experimenter near their sitting positions to allow them to give a “ready” signal during the measurement process and to reduce the need for verbal communication. All the test surfaces and the test setup were prepared and stored for 3 months in the workshop to avoid any material or treatment-related odors.

### Touch questionnaire

The affective characteristics of touch were measured by “affective descriptors” from the TPT scale (Guest et al., 2011) translated into Finnish. The translation process involved three forward translations followed by three backward translations. After translation, we used fewer numbers of descriptors (Table 2) since some English words were combined into one after Finnish translation and some descriptors were considered to be less important for wood touch. The descriptors “enjoyable and pleasurable” were translated into the single word *nautinnollinen*. “Calming and soothing” have the same meaning in Finnish–*rauhoittava*. “Exciting and thrilling” was also translated into one word – *jännittävä*. Similarly, the descriptors “sexy,” “arousing,” and “sensual” were translated to *seksikäs, kiihottava*, and *aistikas* respectively. Comparing the results from the forward and backward translations and their dictionary meanings, the three native Finnish-speaking authors of this paper concluded that these three translated Finnish words (i.e., *seksikäs, kiihottava*, and *aistikas)* did not occur naturally in the expressions of emotional touch in this context. Our decision to exclude these three descriptors from wood touch evaluation is supported by the notion that linguistic differences exist in expressing emotion (Fussell, 2002) and often the original meaning is reduced or lost after translation (for example, see Van Nes et al., 2010). Eight emotional descriptors resulted from this translation process. The scale choice consisted of five points: “not descriptive,” “slightly descriptive,” “moderately descriptive,” “highly descriptive,” and “very highly descriptive.” The scale choices, questionnaire and test instructions were also translated into Finnish. In addition, three sensory descriptors – “rough” (*karkea*), “smooth” (*sileä*), and “slippery” (*liukas*)–were included in the questionnaire.

**Table 2:** **The list of emotional and sensory descriptors used in the experiment**.

**Affective descriptors**						**Sensory descriptors**		
	**English**	**Finnish**		**English**	**Finnish**		**English**	**Finnish**
1.	Irritating	Ärsyttävä	5.	Relaxing	Rentouttava	1.	Rough	Karkea
2.	Comfortable	Miellyttävä	6.	Discomfort	Epämukava	2.	Smooth	Sileä
3.	Enjoyable	Nautinnollinen	7.	Desirable	Haluttava	3.	Slippery	Liukas
	Pleasurable							
4.	Calming	Rauhoittava	8.	Exciting	Jännittävä			
	Soothing			Thrilling				

### Procedure

During the experiment, participants were asked to sit comfortably in front of the test setup and practice the mode of touch. A native Finnish speaker gave all the instructions in Finnish. The participants were asked to wear headphones to avoid any auditory disturbances. The eight wood stimuli and two reference stimuli (Table 1) were presented randomly during each block of presentations (Table 3). The stimuli sequences were designed in such a way that the reference stimuli were not presented first or last. In addition, they did not appear in succession; at least two wood stimuli appeared between them. All 11 descriptors were placed randomly in the questionnaire and presented in a different order for each trial. During the trials, each participant was asked to move their four fingertips (excluding thumb) laterally over the stimulus surface at a speed of ~3 cm/s, making four transverse movements totaling 24 cm and traveled within 7–9 s. A figure illustrating the hand movement was placed in front of the participant as a constant reminder throughout the measurement. Touch pressure was not controlled, but the participants were instructed to practice uniform finger speed and touch pressure for 5–6 min over A4 paper before the data collection began.

**Table 3:** **Pearson correlation between touch descriptors**.

	**Irritating**	**Comfortable**	**Pleasurable**	**Calming**	**Exciting**	**Relaxing**	**Desirable**	**Discomfort**	**Rough**	**Smooth**
Comfortable	**−0.658**									
Pleasurable	**−0.665**	**0.902**								
Calming	**−0.589**	**0.877**	**0.848**							
Exciting	0.004	**0.196**	**0.256**	**0.170**						
Relaxing	**−0.624**	**0.881**	**0.882**	**0.919**	**0.173**					
Desirable	**−0.508**	**0.801**	**0.847**	**0.753**	**0.290**	**0.785**				
Discomfort	**0.828**	**−0.683**	**−0.685**	**−0.649**	0.042	**−0.681**	**−0.533**			
Rough	**0.156**	0.047	0.040	0.039	**0.416**	0.051	**0.129**	**0.227**		
Smooth	−0.069	**0.330**	**0.279**	**0.297**	**−0.331**	**0.297**	**0.244**	**−0.258**	**−0.484**	
Slippery	**−0.339**	**0.539**	**0.488**	**0.498**	−0.048	**0.486**	**0.358**	**−0.473**	**−0.238**	**0.638**

*Pearson correlation coefficient. Significant values sought at 0.05 level are presented in bold*.

Once the participant was ready for the trial, they first rang the bell to give the ready signal and then the experimenter moved the stimulus into position and responded by giving another ready signal, informing the participant that the stimulus was ready to touch. The participant then touched the exposed stimulus for 8 s, and rated the 11 descriptors immediately using paper and pencil. Ten stimuli (eight wood stimuli and two reference stimuli) were rated in one block. This task was then repeated four times (i.e., in total, there were four blocks in which the ten stimuli were presented). Each block was followed by a short break before the next block commenced. The order in which the stimuli were presented within the block was randomized and likewise the lists of descriptors in the questionnaire.

### Data analysis

Statistical analysis was carried out using SPSS (version 23; IBM). The participants' scores on all the descriptors for each stimulus were averaged from the four trials and descriptive statistics from the two reference samples were calculated separately for comparison purposes. For the rest of the data from the wood samples, the results were investigated using one-way ANOVA to check for any gender effect on each descriptor rating, but no significant gender effects were observed. Multivariate analysis of variance (MANOVA) was used to test the effect of wood type and surface treatment conditions in each affective and sensory descriptor. The wood condition had two levels (pine and oak), and surface treatment condition had four levels (sanded, brushed, varnished, and waxed). Histograms and boxplots were used to check for any outliers under different levels and conditions prior to MANOVA and moderate to high levels of negative and positive (Pearson) correlations were found between the measured variables (see Table 3). Separate MANOVA tests were conducted using the three sensory descriptors and eight affective descriptors as dependent variables, adopting a full factorial model. Box's Test was used to test the equality of covariance metrics and Levene's Test was used to test the homogeneity of variance for all dependent variables. Pillai's Trace *F*-Test was selected as the most robust method for equal group size among multivariate test statistics (Field, 2013). Separate univariate ANOVAs on outcome variables (tests of between-subject effects) were also calculated to see the effect of each condition on dependent variables. A Bonferroni-corrected post hoc test was used for multiple comparisons between different levels under each condition if the main effect was found to be significant. Statistical significances were sought at *p* < 0.05.

## Results

**Figure 3:**
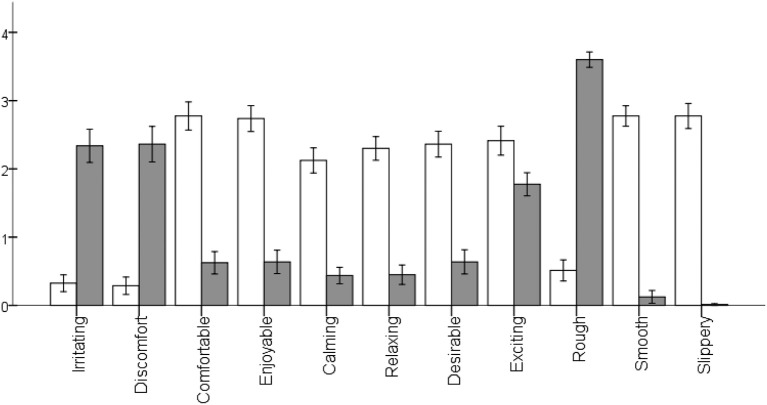
**Average scores of sensory and emotional descriptors in the evaluation of silk cloth and sandpaper-240**. Error bar represents ±1 SD. *Scale: 0* = “*not descriptive*,” *4* = “*very highly descriptive*.”

As detailed above, the focus of this experiment was an evaluation of the sensory and emotional touch descriptors of various natural and surface-treated wood surfaces in an active fingertip exploration mode among Finnish participants. An analysis of the evaluation of the emotional and sensory descriptors for reference stimuli (sandpaper and silk cloth) showed that the participants were able to perceive them in the same way as reported in previous studies (sandpaper in Ackerley et al., 2014a and silk cloth in Guest et al., 2011). Figure 3 shows that these stimuli were evaluated at extreme boundary conditions in all the sensory and emotional attributes (i.e., both reference stimuli were rated either toward the higher end or toward the lower end of the scale, except for “exciting” where both were rated at a moderate level).

**Figure 4:**
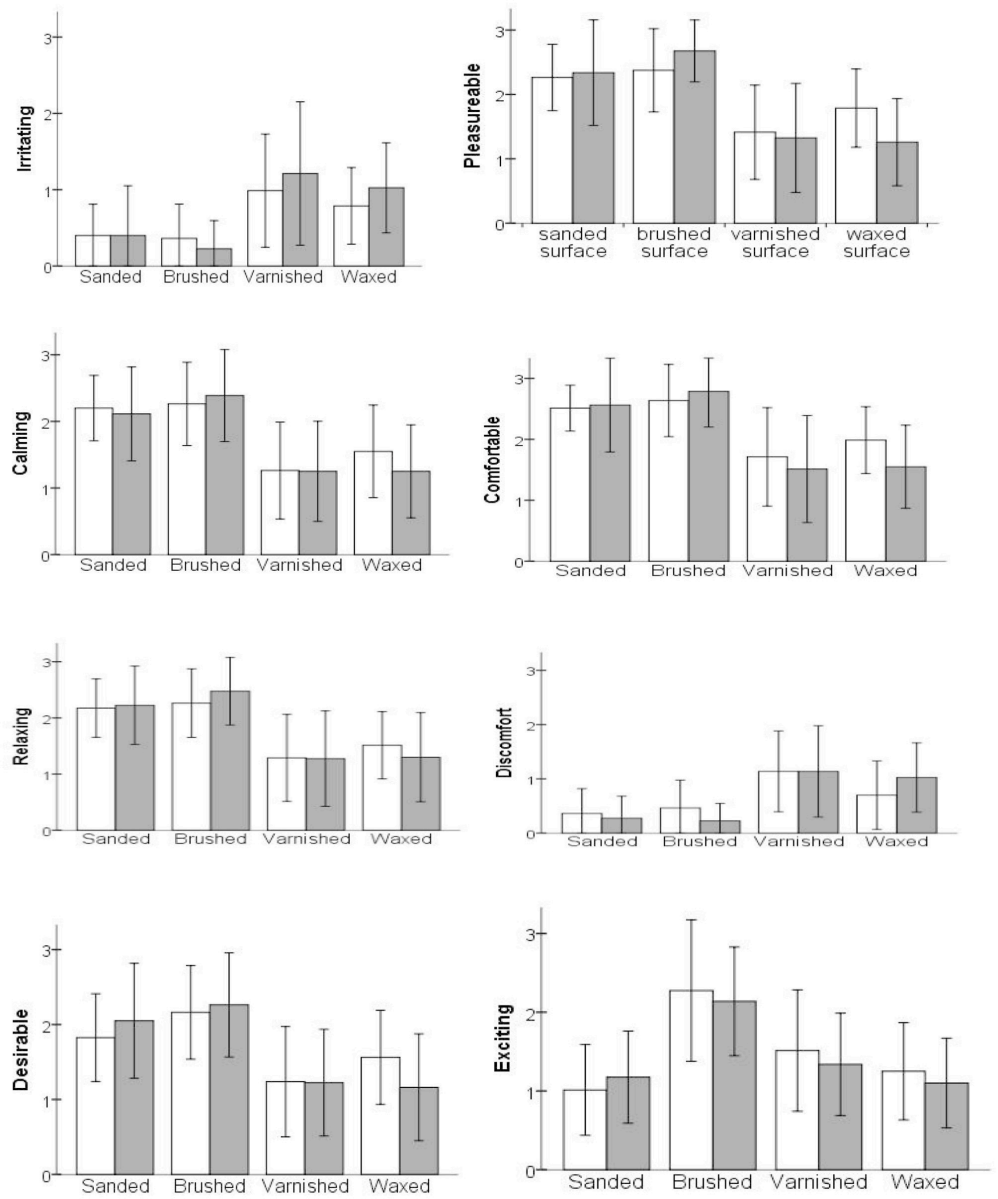
**The average scores for emotional descriptors**. Each descriptor is displayed with the average scores for the wood type condition under four different surface treatment conditions. Error bar represents ±1 SD. *Scale: 0* = “*not descriptive*,” *4* = “*very highly descriptive*.” For details of significant differences, see Table 4.

Multivariate analysis of variance (MANOVA) of the data revealed differences in the perception of the affective and sensory components of the tactile experiences with regard to the various surface-treatment methods applied to the two types of wooden surfaces (pine and oak wood). In the MANOVA tests with affective descriptors as dependent variables, a significant main effect of the surface treatment condition was found, but wood type showed no effect. The univariate results suggest that the surface treatment effect is significant on all the measured affective descriptors (Table 4). *Post hoc* comparison results show that there was no significant difference between uncoated surfaces (i.e., between sanded and brushed surfaces) on the emotional perception except for “exciting,” where the brushed surfaces received higher ratings than the sanded surfaces. It is interesting that both coated surfaces were evaluated in a similar fashion on all emotional descriptors. However, both uncoated surfaces were perceived to be significantly different to the coated surfaces (i.e., varnished and waxed surfaces). Post hoc comparison shows that the coated surfaces were rated significantly higher in terms of “irritating” and “discomfort,” whereas they were significantly lower in other emotional ratings (Table 4; and for mean differences, see Figure 4). Some inconsistencies were observed with the perception of the “exciting” attribute of the wooden surfaces, as it was not linked to the ratings of the negative or the positive aspects of the emotional descriptors. The brushed surfaces scored the highest ratings, whereas the varnished and waxed surfaces were rated equally exciting. However, the sanded and varnished surfaces appeared to be equally exciting, and the waxed surfaces were significantly more so than the sanded surfaces.

**Table 4:** **Significant differences for the condition type in the affective descriptors**.

**Conditions**	**Affective Descriptors**							
	**Irritating**	**Comfortable**	**Pleasurable**	**Calming**	**Relaxing**	**Discomfort**	**Desirable**	**Exciting**
Wood Type	*F*_(1, 159)_ = 0.713	*F*_(1, 159)_ = 1.085	*F*_(1, 159)_ = 0.318	*F*_(1, 159)_ = 0.412	*F*_(1, 159)_ = 0.007	*F*_(1, 159)_ = 0.001	*F*_(1, 159)_ = 0.041	*F*_(1, 159)_ = 0.488
*F*_(8, 145)_ = 0.802 n.s.	n.s.	n.s.	n.s.	n.s.	n.s.	n.s.	n.s.	n.s.
Surface Treatments	*F*_(3, 159)_ = 16.393	*F*_(3, 159)_ = 26.552	*F*_(3, 159)_ = 28.238	*F*_(3, 159)_ = 24.403	*F*_(3, 159)_ = 25.454	*F*_(3, 159)_ = 18.429	*F*_(3, 159)_ = 18.511	*F*_(3, 159)_ = 22.364
*F*_(24, 441)_ = 5.523, *P* < 0.001	*P* < 0.001	*P* < 0.001	*P* < 0.001	*P* < 0.001	*P* < 0.001	*P* < 0.001	*P* < 0.001	*P* < 0.001
Sanded–Brushed	n.s.	n.s.	n.s.	n.s.	n.s.	n.s.	n.s.	*P* < 0.05
Sanded–Varnished	*P* < 0.001	*P* < 0.001	*P* < 0.001	*P* < 0.001	*P* < 0.001	*P* < 0.001	*P* < 0.001	n.s
Sanded–Waxed	*P* < 0.001	*P* < 0.001	*P* < 0.001	*P* < 0.001	*P* < 0.001	*P* < 0.001	*P* < 0.001	*P* < 0.001
Brushed–Varnished	*P* < 0.001	*P* < 0.001	*P* < 0.001	*P* < 0.001	*P* < 0.001	*P* < 0.001	*P* < 0.001	*P* < 0.001
Brushed–Waxed	*P* < 0.001	*P* < 0.001	*P* < 0.001	*P* < 0.001	*P* < 0.001	*P* < 0.001	*P* < 0.001	*P* < 0.001
Varnished–Waxed	n.s.	n.s.	n.s.	n.s.	n.s.	n.s.	n.s.	n.s.

*For each condition (wood type and surface treatments), the main effect is given in F-values with degrees of freedom and highlighted in shading. The effect of each condition on each affective descriptor (between-subjects effects) are given in F-values with degrees of freedom under corresponding descriptors. If a significant main effect of conditions was found, Bonferroni-corrected post hoc comparisons were made. Probability (p) values for significant difference were given as P < 0.001, P <0.01, and P < 0.05. n.s. denotes no significant differences. (for details on mean differences, see Figure 4)*.

**Figure 5:**
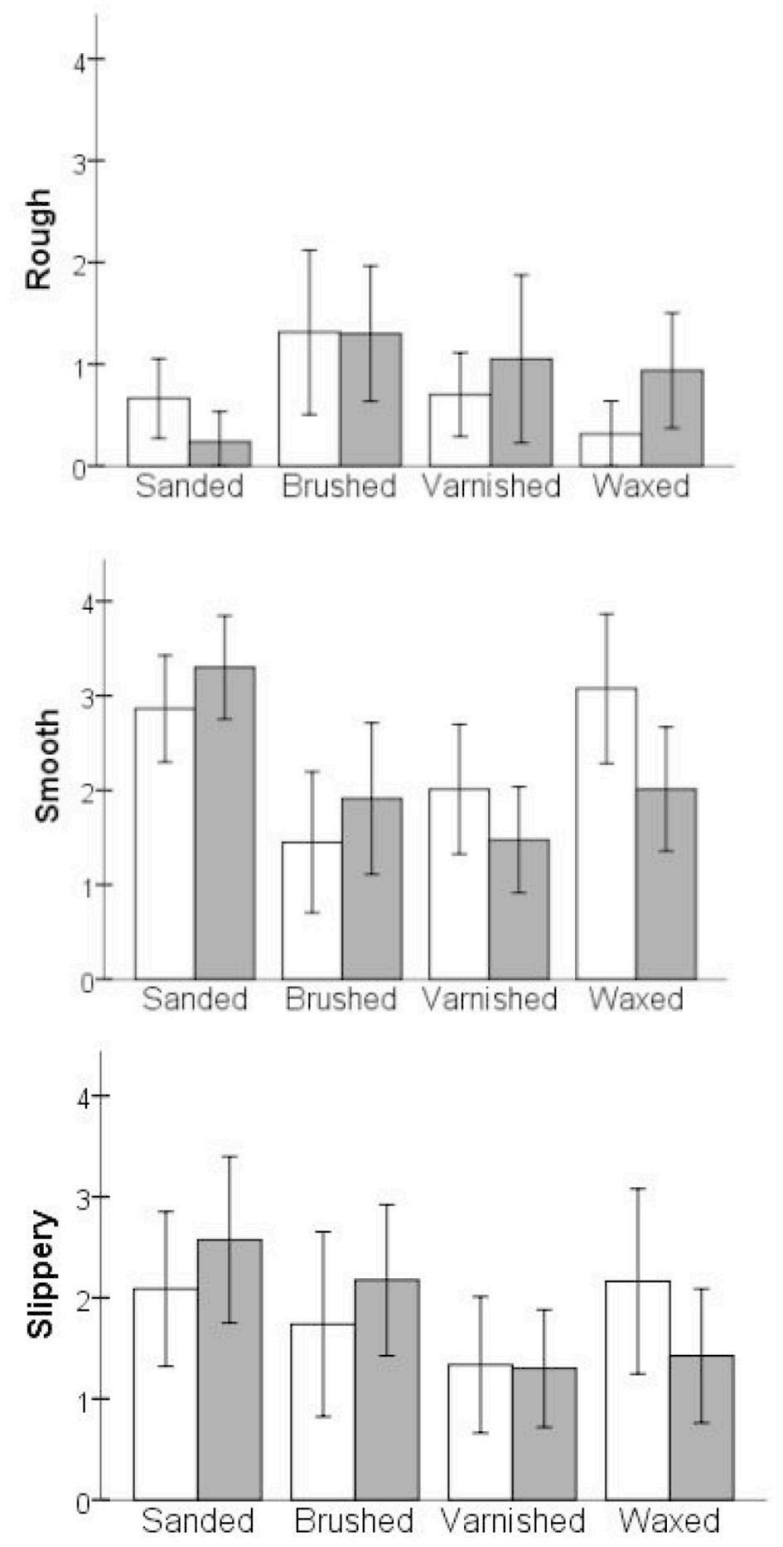
**The average scores for sensory descriptors. Each descriptor is displayed with the average scores for the wood type condition under four different surface treatment conditions**. Error bar represents ±1 SD. *Scale: 0* = “*not descriptive*,” *4* = “*very highly descriptive*.” For details of significant differences, see Table 3.

Similarly, in the MANOVA test with the three sensory descriptors as dependent variables, the main effect of the surface treatment was significant but the effect of the wood type was not (Table 5). Further, results from univariate ANOVA tests suggest that the effect of the surface treatment method was significant on all of the three sensory descriptors. Amongst the natural surfaces, *post hoc* comparison revealed that the sanded surfaces were rated significantly lower in terms of roughness, higher in smoothness and higher in slipperiness than the brushed surfaces (Table 5; and for mean differences, see Figure 5). Comparing the sensory ratings of the coated surfaces, the waxed surfaces were rated higher in terms of smoothness and slipperiness than the varnished surfaces, but they appeared to be similar in the roughness evaluation. Further, comparing the natural and coated surfaces in the sensory evaluation, the sanded surfaces were perceived to be higher in terms of smoothness and slipperiness than the varnished and waxed surfaces, but lower or equal in the roughness perception than the varnished and waxed surfaces, respectively. Additionally, the brushed surfaces were perceived to be higher in terms of roughness, lower in smoothness but similar in slipperiness compared to the waxed surfaces. To summarize, all four types of treatment applied to the wood surfaces had unique effects on discriminative sensation.

**Table 5:** **Significant differences for the condition type in the sensory descriptors**.

**Conditions**	**Sensory descriptors**		
	**Rough**	**Smooth**	**Slippery**
Wood Type	*F*_(1, 159)_ = 2.211	*F*_(1, 159)_ = 2.680	*F*_(1, 159)_ = 0.095
*F*_(3, 150)_ = 2.38, ns	n.s.	n.s.	n.s.
Surface Treatment	*F*_(3, 159)_ = 16.910	*F*_(3, 159)_ = 39.561	*F*_(3, 159)_ = 11.893
*F*_(9, 456)_ = 15.217, *P* < 0.001	*P* < 0.001	*P* < 0.001	*P* < 0.001
Sanded–Brushed	*P* < 0.001	*P* < 0.001	*P* < 0.05
Sanded–Varnished	*P* < 0.001	*P* < 0.001	*P* < 0.001
Sanded–Waxed	n.s.	*P* < 0.001	*P* < 0.01
Brushed–Varnished	*P* < 0.01	n.s.	*P* < 0.01
Brushed–Waxed	*P* < 0.001	*P* < 0.001	n.s.
Varnished–Waxed	n.s.	*P* < 0.001	*P* < 0.05

For each condition (wood type and surface treatments), the main effect is given in F values with degrees of freedom and highlighted in shading. The effect of each condition on each sensory descriptor (between-subjects effects) are given in F values with degrees of freedom under corresponding descriptors. If a significant main effect of conditions was found, Bonferroni-corrected post hoc comparisons were made. Probability (p) values for significant difference were given as P < 0.001, P < 0.01 and P < 0.05. n.s. denotes no significant differences. (for details on mean differences, see Figure 5)

## Discussion

In this investigation, we explored the sensory and emotional touch evaluation of various wooden surfaces through the lateral motion of active fingertip exploration. Eight wood surfaces along with one silk and one sandpaper were rated using a Finnish version of the emotional descriptors in the TPT. Significant differences in perception due to the effect of the surface treatment methods were observed between natural and coated wood surfaces. Natural wood surfaces were rated significantly higher in all the descriptors that feature positive aspects of the emotional component. Additionally, the natural surfaces were rated least irritating and uncomfortable, descriptors that feature the negative aspects of affective touch. The trends in emotional ratings identified in previous TPT studies (Guest et al., 2011; McGlone et al., 2012; Ackerley et al., 2014a) were closely mirrored in our results, and are interesting from a manufacturing as well as research perspective.

The natural surfaces were perceived to be significantly different in all of the three sensory descriptors. However, they scored similar ratings in all the emotional descriptors except for “exciting.” Some studies suggest that smooth, slippery, and soft items are more pleasant to touch than rough and sticky items (Essick et al., 1999, 2010). Klöcker et al. (2012) tested 58 different materials including sandpaper, silk and wood through active fingertip exploration, where sandpaper was perceived to be very high in the roughness and very low in the pleasantness perception, and silk appeared to be in the group consisting of the most pleasant to touch materials that were smooth, which also agrees with our results from the reference stimuli. They also found that the pleasantness perception depends on the roughness and stickiness of hard material surfaces like wood, tile, Plexiglas and marble. However, we found that smoother wood surfaces could elicit a higher pleasantness or other positive emotional rating, but rough surfaces had no effect on it. This undermines some prior findings on sensory-emotional touch relations such as the existence of an inverse correlation between roughness and pleasantness (Essick et al., 2010; Klöcker et al., 2012). We tested a narrow range of roughness among wood stimuli, but this range is far higher than the roughness magnitudes normally applied to the surfaces of commercial wood products, such as furniture, walls and floors. Roughness showed a moderate level of positive correlations with both touch descriptors that feature negative aspects of touch descriptors–irritation and discomfort–which is in line with previous findings on the factors contributing to the unpleasantness perception (Essick et al., 1999, 2010; Klöcker et al., 2012). It would be interesting to direct future research to see whether the surface roughness in commercial wood products is important for the pleasant-to-touch experience, or whether other textural properties like smoothness and slipperiness play a role.

Further results show moderate-to-high positive correlations between slipperiness and all the measured positive aspects of emotional touch, which are similar to previous findings in pleasant-touch evaluation studies (Essick et al., 2010; Klöcker et al., 2012). The negative correlations between slipperiness and the other two negative aspects of emotional touch (irritation and discomfort) are also in line with previous results, where the less slippery materials such as sandpaper, latex and wax were rated higher in unpleasant to touch (Guest et al., 2011; Klöcker et al., 2012; Ackerley et al., 2014a). It is interesting that slipperiness normally remains a secondary dimension in psychophysical studies (Okamoto et al., 2013) though we noted a considerable influence on the emotional touch evaluation of wood surfaces. The presence of lower magnitudes of roughness among wood stimuli would explain this influence, but it would still be interesting to see the effect of slipperiness on the emotional touch of other similar surfaces/products where the roughness level is often narrow.

Although the coated surfaces in general appeared to be distinct from each other in sensory ratings, they were evaluated similarly in emotional ratings. For example, the varnished surfaces appeared to be the least smooth amongst the coated surfaces and the least slippery among all wood stimuli. This could be explained by the product-specific effect of varnishing wooden surfaces, namely that varnish forms a film (unlike wax) on the wood surface. These types of coatings are the most common surface treatment methods in the wood industry to improve durability, hydrophobicity and aesthetic quality. The lower positive emotional ratings (pleasant-to-touch etc.) for all the coated surfaces cautions against compromising the consumer's positive touch experience when making efforts to engineer technical improvements in wood. It seems that retaining the naturalness of wooden surfaces, after applying treatments, remains important in improving touch experiences. Additionally, many kinds of wood surface treatment methods exist which require a variety of surface coating procedures; how these coatings affect the affective touch perception of wooden surfaces would be an interesting topic for further investigation.

Another important feature of wood is that it is never possible to create two identical surfaces–there will always be variations in the surface due to, for example, anatomical structure, knots, the presence or absence of checks, and the grain pattern on the surface. Using the same type of sandpaper does not guarantee that the two surfaces will be identical, but it is possible to create similar surfaces from a single wood species by selecting surfaces that have similar physical characteristics first, and then using the same sanding procedure. It was not even possible to make wood surfaces very rough because the properties of the wood fibers vary from species to species. This is also one of the reasons for including sandpaper as a reference stimulus in the experiment to give an upper extreme boundary condition of roughness. The effect of fingertip force on the discriminatory sensation during exploratory (active) touch, however, has not been studied extensively (see Taylor and Lederman, 1975 for roughness perception), but a gentle, light touch on the skin with a moderate brush stroking velocity (here, fingertip speed over wood surfaces) would be required to feel an optimal level of pleasantness (Löken et al., 2011; Perini et al., 2015). In this study, we asked participants to use a uniform finger speed of about 3 cm/s with a light touch pressure during the experimentation but did not measure the touch force. The reason for not observing an effect due to wood species, in both types of touch evaluation (sensory and emotional), might be the existence of relatively small intrinsic differences in the essence of the tactual properties of the wood (Bergmann Tiest, 2010), like grooves and ridge height/width etc., rather than being due to the light fingertip force applied during the touch.

Previous studies have shown that, in general, fingertip touch is very good at the sensory evaluation of surface properties due to the higher discriminatory ability of the A-beta afferent neurons present in glabrous skin compared to hairy skin (Essick et al., 2010) in both active and passive touch (Verrillo et al., 1999; Guest et al., 2011; Ackerley et al., 2014a). We observed a considerable amount of consistency across the participants' responses to the measured sensory descriptors across varieties of wood stimuli. Results from the present study also support the fact that the glabrous fingertip performs very well in the evaluation of the emotional aspects of touch even though it lacks C-tactile afferent neurons which are believed to be responsible for the emotional sensation in hairy skin (Löken et al., 2009, 2011; Klöcker et al., 2012; McGlone et al., 2012; Ackerley et al., 2014a). We would argue, therefore, that the higher accuracy in discriminative sensation and the greater possibilities for fingertip touch experiences leads to higher affective responses whether positive or negative.

The pleasantness and related emotional touch responses have shown strong associations with reward-related evaluation and decision-making behavior (Perini et al., 2015) and are also of interest in product design and in market research (for a review, see Krishna, 2012). Therefore, emotional touch lead by the fingertip is much more likely to influence both planned (deliberate) and unplanned (impulsive) consumer behavior. Our results suggest the possibility of improving emotional touch experiences, especially of wooden products, and ultimately influencing the touch-based decision-making behavior in, for example, impulse buying (Peck and Childers, 2006).

## Conclusion

In this study, we investigated emotional touch perception through an active fingertip exploration across varieties of pine and oak wood surfaces. The present study showed that the effects of coatings applied to the various wooden surfaces had a significant association with the affective evaluation, especially with positive aspects. These findings anticipate the possibility of improving the comfort-touch of wood-based products. Retaining the naturalness of the surface properties of wood-based products seems to be crucial for improving positive touch experiences and thereby influencing decision-making behavior.

## Ethics statement

This study was carried out in accordance with the recommendations of “Aalto Univeristy Research Ethics Committee” with written informed consent from all subjects. All subjects gave written informed consent in accordance with the Declaration of Helsinki.

## Author contributions

All authors contributed to the conception of the work, design of the experiment and critical revision of the draft. The authors SB and KV carried out the data collection. SB and KT analyzed and interpreted the data. All authors participated in writing, approved the final version and agree to be accountable for all aspects of the work.

## Funding

This work was supported by the Wood Life project [2013–2017] funded by Aalto University within the framework of the Aalto Energy Efficiency Research Programme (AEF).

### Conflict of interest statement

The authors declare that the research was conducted in the absence of any commercial or financial relationships that could be construed as a potential conflict of interest.
